# Identification of a Cowden syndrome patient with a novel *PTEN* mutation and establishment of patient-derived induced pluripotent stem cells

**DOI:** 10.1007/s11626-021-00637-8

**Published:** 2022-01-03

**Authors:** Fumitaka Obayashi, Atsuko Hamada, Sachiko Yamasaki, Taku Kanda, Shigeaki Toratani, Tetsuji Okamoto

**Affiliations:** 1 Department of Oral and Maxillofacial Surgery, Hiroshima University Hospital, Hiroshima, Japan; 2 Department of Oral and Maxillofacial Surgery, Hiroshima Prefectural Hospital, Hiroshima, Japan; 3 Department of Molecular Oral Medicine and Maxillofacial Surgery, Graduate School of Biomedical and Health Sciences, Hiroshima University, 1-2-3 Kasumi, Minami-ku, Hiroshima, 734-8553 Japan; 4 School of Medical Sciences, The University of East Asia, Yamaguchi, Japan

**Keywords:** Cowden syndrome, *Phosphatase and tensin homolog deleted on chromosome 10*, *Phosphatase and tensin homolog deleted on chromosome 10 δ*, Induced pluripotent stem cells, Feeder- and serum-free culture condition

## Abstract

Cowden syndrome (CS) is an autosomal dominant inherited disorder characterized by multiple hamartomas in various organs such as the mucosa, skin, and gastrointestinal tract. Patients with CS are at high risk for breast and thyroid cancers. *Phosphatase and tensin homolog deleted on chromosome 10* (*PTEN*) is a tumor suppressor gene that negatively regulates the AKT pathway, and *PTEN* mutations are known to be the major causes of this syndrome. However, the pathogenesis of this syndrome has not been clarified. Here, we present a case of a Japanese woman with multiple oral polyps, breast cancer, and thyroid cancer who was clinically diagnosed with CS. We obtained DNA and RNA samples from the patient’s peripheral blood mononuclear cells (PBMCs) and buccal mucosa tumor. Next-generation sequencing revealed novel germline mutations (c.1020delT and c.1026G > A) in exon 8 of *PTEN*. Sanger sequencing identified no *PTEN* transcript from the mutant allele. Furthermore, CS-specific induced pluripotent stem cells (CS-iPSCs) were established from PBMCs of the patient under feeder- and serum-free culture. Compared with healthy PBMCs and iPSCs, both of the CS-derived PBMCs and CS-iPSCs exhibited significantly reduced expression of the *PTEN* transcript. The transcriptional variant, PTEN*δ*, was increased in CS-iPSCs, suggesting that it may be the cause of the disease.

**Supplementary Information:**

The online version contains supplementary material available at 10.1007/s11626-021-00637-8.

## Introduction

Cowden syndrome (CS) (MIM 158,350) is a disease that causes hamartoma neoplasms in multiple organs, including the skin, mucous membranes, and gastrointestinal tract (Eng, 1997). It is a rare autosomal dominant genetic disorder that occurs in 1 per 250,000 people. Throughout their lifetime, CS patients have an increased risk of developing breast and thyroid cancer, specifically 50–85% and 30–40%, respectively (Tan *et al.*, 2012). Because of the high risk of developing cancer, the National Comprehensive Cancer Network (NCCN) clinical practice guidelines in the U.S.A. recommend CS patients undergo careful medical testing and genetic counseling (NCCN.org, 2019). It is known that most cases of CS result from a mutant *phosphatase and tensin homolog deleted on chromosome 10 (PTEN)* allele (MIM 601,728) (Eng, 2000). *PTEN* is well known as a major tumor suppressor gene. The PTEN protein is a multifunctional phosphatase that regulates the AKT/mTOR pathway and plays an important role in the regulation of cell behavior, including growth, survival, and migration (Milella *et al.*, 2015). *PTEN* mutations are detected in many malignant neoplasms. However, since there are few studies on the relationship between *PTEN* mutations and the phenotype of CS, the pathogenesis of this disease remains unclear.

Since Takahashi and Yamanaka reported the establishment of induced pluripotent stem cells (iPSCs) in mice (Takahashi and Yamanaka, 2006), iPSCs have been established from patients with many hereditary diseases and have been used to elucidate their pathogenesis and develop therapies. In the field of oncology, the use of iPSCs with the genetic backgrounds of patients with hereditary tumor syndromes may lead to the elucidation of not only the development of hereditary tumors but also the mechanism of somatic carcinogenesis. We have previously reported the induction of iPSCs from dental pulp and peripheral blood mononuclear cells (PBMCs) of patients with several genetic disorders under feeder-, serum-free culture conditions (Yamasaki *et al.*, 2014; Hamada *et al.*, 2020a, 2020b). In the present study, we found two novel *PTEN* exon 8 mutations in the same allele in a Japanese CS patient. In addition, we established disease-specific iPSCs from PBMCs of CS patients and investigated the expression of the *PTEN* gene and *PTENδ* splice variant in CS-PBMCs and CS-iPSCs.

## Materials and methods

### **Ethics statement**

This study was approved by the Ethics Committee of Human Genome-Gene Analysis Research at Hiroshima University (approval number: hi-58 and hi-72). All animal experiments in this study strictly followed the protocol approved by the Institutional Animal Care and Use Committee of Hiroshima University (approval number: A-11–140).

### **Phenotype of the affected individual**

In 2014, a 60-yr-old Japanese woman presented to our Department of Oral Maxillofacial Surgery at Hiroshima University Hospital with multiple oral polyps and pain in the buccal mucosa upon biting. She had first noticed oral polyps at the age of 25 yr. Eventually, these polyps extended to the whole mouth, and she sought care in otolaryngology. She had an extensive medical history: uterine fibroid at age 24, systemic scleroderma at age 37, thyroid cancer at age 41, a gastric lymphatic polyp at age 43, breast cancer at age 45, rheumatoid arthritis at age 53, and pericarditis pleurisy at age 55. She was treated for these diseases and received follow-up care in the hospital. However, she was never diagnosed with a syndrome, and there was no evident family history related to her condition.

The patient had a height of 158 cm, weight of 61.8 kg, and a body mass index of 24.7. Her head circumference was 58 cm (97th percentile), and she had macrocephaly. Many small mucosal papules appearing as “cobblestones” were present on the tongue, upper lower lip, palate, gingiva, and buccal mucosa. Each tumor was 2–8 mm in diameter, elastic, soft, and with a smooth surface. Endoscopic examination revealed a papillomatous lesion in the upper and lower gastrointestinal tract. Her face was dotted with trichilemmomas and hyperkeratotic papules (Supplementary data 1A-E). Histological examination of the excised buccal tumor tissue confirmed warty mucosal proliferation (Supplementary data 1F-G). Neither acral keratosis nor intellectual disability was observed. We clinically diagnosed her with CS based on the syndrome-testing criteria adapted by NCCN.

### **Genetic analysis**

We obtained written informed consent from the patient for genetic testing. Her DNA was extracted from PBMCs using a QIAamp® DNA mini kit (Qiagen, Valencia, CA), according to the manufacturer’s instructions. PBMC samples were prepared by centrifugation in a Histopaque-1077 (Sigma Aldrich, St. Louis, MO) density gradient.

### **Mutation analysis using next-generation sequencing (NGS)**

To detect pathogenic mutations, targeted sequencing was performed with the MiSeq System and the TruSight One Panel (Illumina, San Diego, CA), which is designed to comprehensively sequence more than 4800 disease-associated genes, according to the manufacturer. We analyzed these data using Illumina VariantStudio 3.0 and the Integrative Genomics Viewer (Broad Institute, Cambridge, MA).

### **Sanger sequencing**

Gene-specific primers were designed in Primer3 (http://bioinfo.ut.ee/primer3-0.4.0/) (Table 1). The polymerase chain reaction (PCR) product was purified with the Wizard SV Gel and PCR Clean-Up System (Promega, Madison, WI) according to the manufacturer’s protocol and sequenced using a CEQ8000 Beckman System (Beckman-Coulter, Brea, CA).

**Table 1:** Primer sequence.

Gene name	Locus	Primer sequence
PTEN DNA	Exon8 Left	5′-AGCGTGCAGATAATGACAAGGA-3′
	Intron8 Right	5′-ACATACAAGTCAACAACCCCC-3′
	Exon7F	5′-TCCACAAACAGAACAAGATGC-3′
	Exon9R	5′-TGCTGATCTTCATCAAAAGGTTC-3′
PTEN cDNA	3′ UTR-F	5′-GTTTACCGGCAGCATCAAAT-3′
	3′ UTR-R	5′-CCCCCACTTTAGTGCACAGT-3′
PTEN δ	Intron8R	5′-ACACACATCACATACATACAAG-3′
OCT3/4	F	5′-GACAGGGGGAGGGGAGGAGCTAGG-3′
	R	5′-CTTCCCTCCAACCAGTTGCCCCAAAC-3′
	F	5′-CAGCCCTGATTCTTCCACCAGTCCC-3′
NANOG	R	5′-CGGAAGATICCCAGTCGGGTCACC-3′
	F	5′-GGGAAATGGGAGGGGTGCAAAAGAGG-3′
SOX2	R	5′-TGCGTGAGTGTGGATGGGATTGGTG-3′
	F	5′-CATGTACGTTGCTATCCAGGC-3′
*β*-ACTIN	R	5′-CTCCTTAATGTCACGCACGAT-3′

### **Reverse transcription-PCR (RT-PCR)**

Total RNA from PBMCs and the buccal mucosa tumor was prepared using TRIzol® Reagent (Thermo Fisher Scientific, Waltham, MA) and the Illustra RNAspin Mini RNA Isolation Kit (GE Healthcare, Chicago, IL), following the manufacturer’s instructions. Isolated RNA was treated with amplification-grade DNase I (Invitrogen, Waltham, MA). RNA was reverse transcribed using the SuperScript VILO cDNA Synthesis Kit (Thermo Fisher Scientific) to synthesize cDNA. A PCR assay was performed using the OnePCR SuperMix (GenedireX Inc., New Taipei City, Taiwan). The products were electrophoresed on a 1.5% agarose gel, and the PCR bands were visualized on the ChemiDoc Touch Imaging System (Bio-Rad, Hercules, CA). Quantitative PCRs (RT-qPCRs) for each sample were carried out on an AriaMx real-time PCR system (Agilent Technologies, Santa Clara, CA), using Brilliant III Ultra-Fast SYBR Green QPCR Master Mix (Agilent Technologies). The expression level of each gene was normalized to the amount of β-actin. The primers used in these experiments are shown in Table 1. The cycle program for product amplification was 1 cycle of 95 °C for 10 min (hot-start activation), followed by 40 cycles of 95 °C for 30 s (denaturation), 55 °C for 1 min (annealing), and 72 °C for 1 min (extension).

### Establishment and characterization of CS-PBMC-derived CS-iPSC

Establishment and maintenance of CS-iPSC under feeder- and serum-free conditions. The establishment of CS-iPSCs from CS-PBMCs (Takahashi *et al.*, 2007) was performed according to a method previously reported under feeder- and serum-free conditions (Hamada *et al.*, 2020a). After culturing PBMCs in serum-free RD6F medium (Sato *et al.*, 1987) supplemented with IL-2 (CELEUK, Takeda Pharm., Osaka, Japan) for 6 d, the cells were infected with Sendai virus vector at MOI = 6 and reseeded on Laminin-E8 in serum-free hESF6 medium supplemented with FGF2, heparin, and activin A (Yamasaki *et al.*, 2014). After 14 d, the embryonic stem (ES) cell–like colonies emerged, colonies were picked, and the cells passaged. Characterization was performed as previously described (Yamasaki *et al.*, 2014).

### **Alkaline phosphatase (ALP) staining**

On day 25 after Sendai virus infection, ALP staining was performed according to the manufacturer’s protocol, using a Fast Red substrate kit (Nichirei Biosciences Inc., Tokyo, Japan). The induction efficiency was calculated as the ratio of the number of ALP-positive colonies to the number of seeded PBMCs.

### **Immunostaining and teratoma formation**

Immunocytochemical analyses of the pluripotency of iPSCs have been described previously (Yamasaki *et al.* 2014). The cells were fixed with 4% paraformaldehyde (PFA) and stained with each antibody against OCT4, NANOG, and SSEA3 (Table 2). The cell nuclei and double-stranded DNA were stained with 4′,6-diamidine-2′-phenylindole dihydrochloride (DAPI). Fluorescence images were captured using a Zeiss inverted LSM 700 confocal microscope (Carl Zeiss GmbH, Jena, Germany). To confirm the in vitro differentiation capacity of CS-iPSCs, we performed an embryoid body (EB)–based three-germ-layer differentiation assay, described previously (Yamasaki *et al.*, 2014). Briefly, undifferentiated CS-iPSCs were cultured in hESF6 medium in low-attachment 96-well plates for 4–5 d, and EBs were then transferred to gelatin-coated 35-mm dishes and further cultured for another 21 d in hESF6, then fixed and stained with each antibody against β III tubulin, αSMA, and AFP (Table 2). *In vivo*, for the teratoma assay, CS-iPSCs were injected into the dorsal flank of SCID (CB17/IcrPrkdcscid/CrlCrlj) mice (1 × 10^6^ cells/100 μL of the cell suspension). Teratomas were harvested approximately 10 wk after the injection; the tumors were surgically dissected. After fixation with PBS containing 4% formaldehyde, teratomas were embedded in paraffin for histology.

**Table 2:** List of antibodies.

Antibody	Cat. no	Company
PTEN	ab32199	Abcam
p-PTEN(Ser380)	#9511	Cell Signaling Technology
AKT	sc-5298	SANTA CRUZ
p-AKT(Ser437)	#9271	Cell Signaling Technology
*β* ACTIN	# 4967S	Cell Signaling Technology
OCT4	MAB4401	Millipore
NANOG	AF1997	R&D
SSEA4	MC813-70	Stemgent
*β* III tubulin	MAB3408/1/637	Millipore
a SMA	N1584	Dako Cytomation
AFP	MAB1368	R & D

### **Western blot analysis**

The iPSCs were added to RIPA buffer containing protease phosphatase inhibitor, and the cells were disrupted with an ultrasonic homogenizer (TOMY SEIKO, Tokyo, Japan). After centrifugation at 13,000 × *g* for 15 min at 4℃, the supernatant was collected and the protein concentration was measured with a Pierce BCA Protein Assay kit (Thermo Fisher Scientific), according to the manufacturer’s instructions. A total of 25 µg of PTEN, phosphorylated PTEN, AKT, and β-actin, and 75 µg of phosphorylated AKT protein were loaded onto a 10% SDS-PAGE gel for electrophoresis. After transferring to PDVF membrane, each antibody was used to detect the appropriate protein (Table 2).

### **Bio-Plex AKT signaling assay**

Twenty micrograms of total protein extracted from each iPSCs was reacted with antibody beads specific for phosphorylated forms of IRS-1, PTEN, AKT, GSK3α/β, BAD, mTOR, p70 S6 kinase, and S6 ribosomal protein and incubated at room temperature for 16 h. Biotinylated antibody was added to the beads for 30 min, followed by addition of streptavidin conjugated phycoerythrin (PE) for 10 min. Phosphoproteins of AKT-Signal Pathway molecules were analyzed by a BioPlex200 system (Bio-Rad).

### **Statistical Analysis**

The data are presented as the mean ± standard error of the mean (SEM) from independent experiments. Statistical analysis included the Student’s *t* test. A *p* value < 0.05 was considered statistically significant.

## Results

### **Novel PTEN genetic mutations were detected by NGS analysis**

**Fig. 1:**
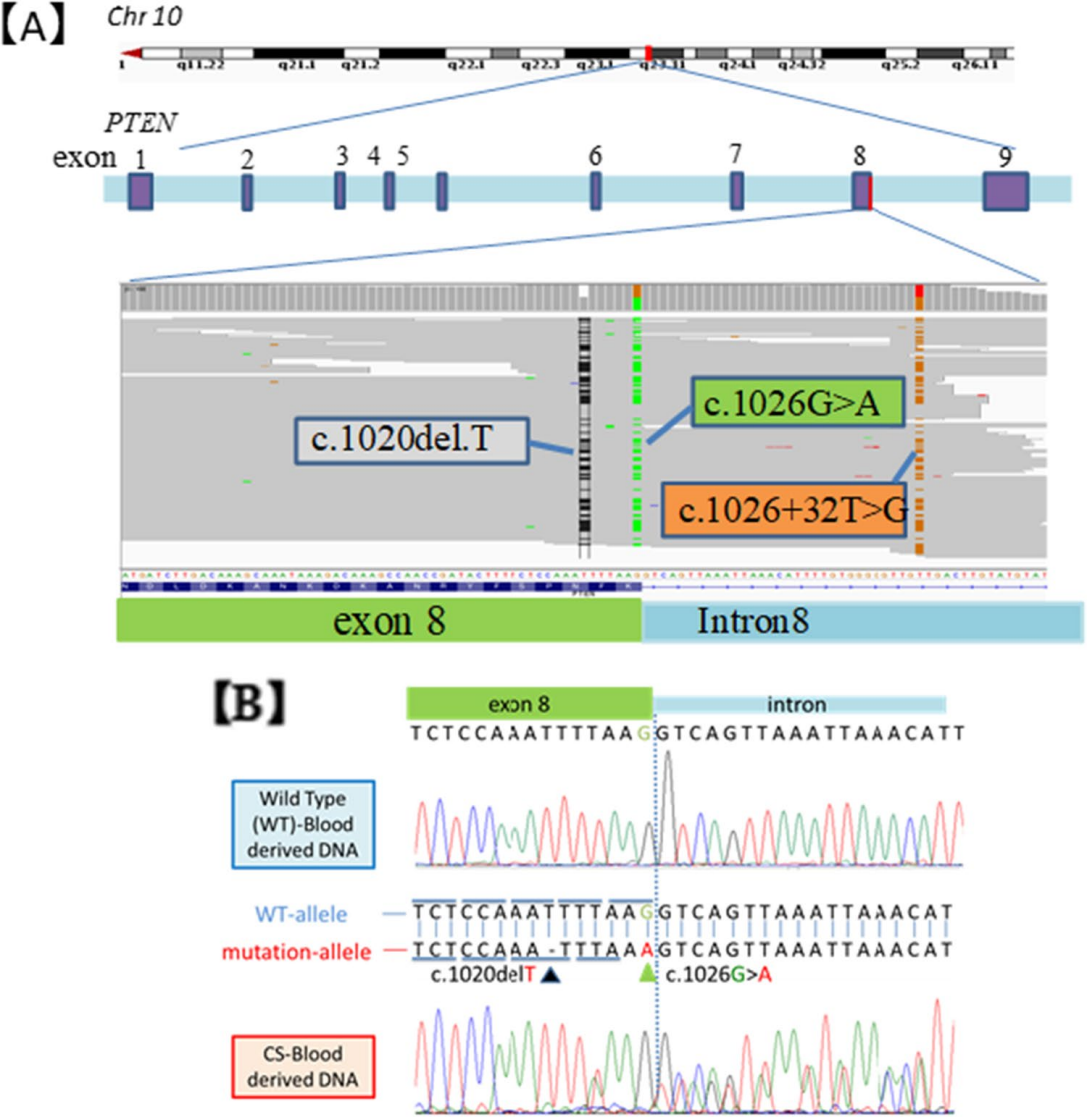
Genetic analysis (*A*, *B*). Mutation of *PTEN* identified by next-generation sequencing (NGS) (*A*) and Sanger sequencing (*B*).

NGS analysis revealed a deletion of base position 1020 T and a mutation of base position 1026 G to A in the coding sequence of the *PTEN* gene (Fig. 1*A*). In addition, c.1026 + 32 T > G mutation was observed. All of these mutations were found on the same read, indicating that they were present in the same allele. The mutations were confirmed by Sanger sequencing (Fig. 1*B*). Moreover, germline mutations of the gene-associated mismatch repair system were detected (Table 3).

**Table 3:** Other genetic mutations detected by next-generation sequencing expected to be involved in this pathology.

Gene	Mutation	Ch	Coordinate	Exonic	Organ	Pathogenicity
MTHFR	G > G/A	1	Missense	5/12	Stomach, colon, acute leukemia	Carcinoma
SAA1	C > C/T	11	Missense	3/4	Rheumatoid arthritis, Crohn’s disease	Carcinoma
BRCA2	A > A/C	13	Missense	10/27	Breast, stomach	Carcinoma
MSH6	G > G/A	2	Missense	1/10	Colon	Carcinoma
HEXB	T > C/C	5	Missense	1/14	Breast	Carcinoma
HYDIN	GA > GA/G	16	Frameshift truncation	69/86	Hematopoietic and lymphoid tissue	Lymphoid neoplasm
MLH1	T > T/A	3	Missense_variant	12/19	Large intestine, pancreas	Carcinoma
TSC2	G > G/A	16	Missense_variant	19/42	Tuberous sclerosis complex	Complex

### **High induction efficiency of CS-iPSCs and their characterization**

**Fig. 2:**
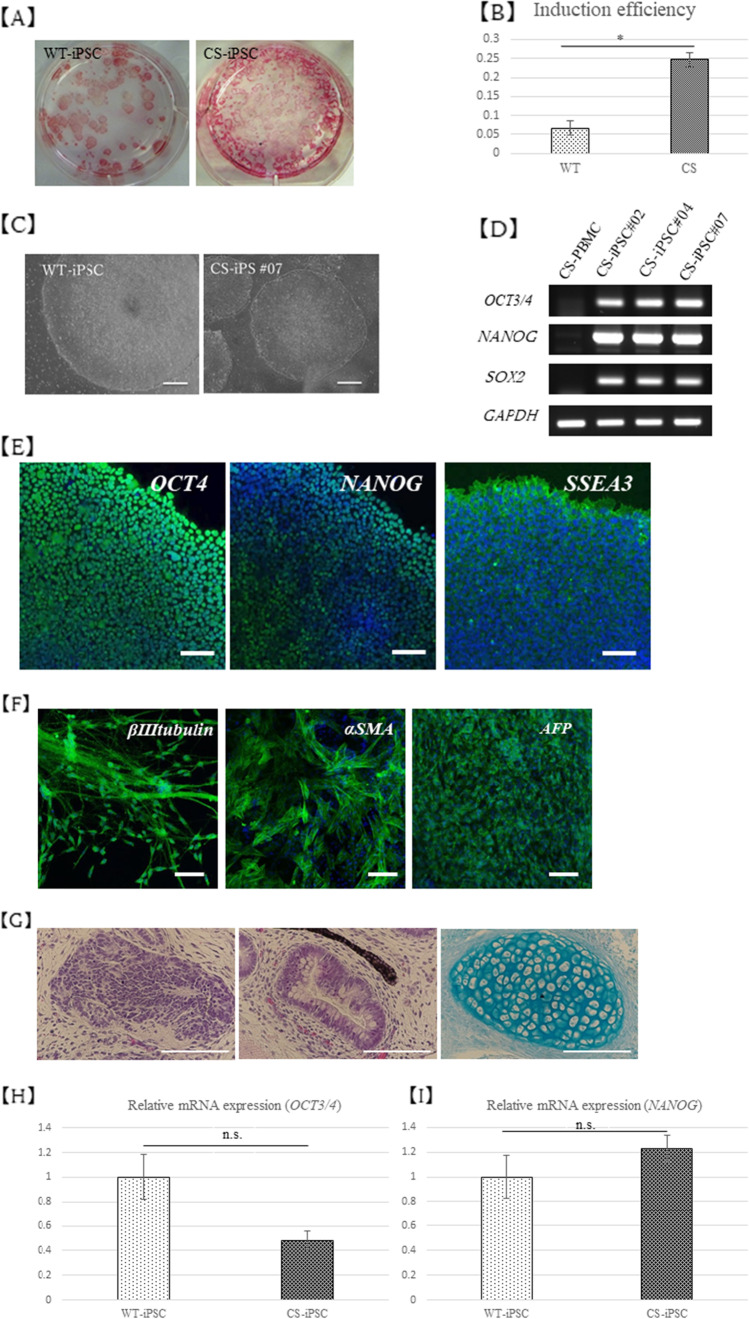
Characterization of the CS-iPSC. (*A*) Alkaline phosphatase staining. (*B*) Induction efficiency of CS-PBMC was higher than that of WT-PBMCs. (*C*) Phase contrast. (*D*, *E*, *F*, *H*, *I*) IF and RT-PCR assay for expression of hES cell surface markers. Each *bar* indicates 100 μm in length. (*G*) *In vivo* teratoma formation assay. Neural tube epithelium (*left*) and intestinal epithelium (*middle*) (H&E stained) and cartilage (*right*) (Alcian Blue-PAS-Stain). Each *bar* indicates 100 μm in length.

After Sendai virus vector infection, ES cell–like colonies appeared in approximately 14 d and could be cultured. The induction efficiency of CS-iPSCs was significantly higher, averaging 0.24%, compared to 0.05% for normal human-derived iPSCs (WT-iPSCs) (Fig. 2*A*, *B*). CS-iPSCs expressed various undifferentiated marker genes and showed the ability to differentiate into three germ layers *in vitro* and *in vivo* (Fig. 2*D*–*G*). However, the expression levels of undifferentiated marker genes and the histological morphology of teratomas were not significantly different from those of WT-iPSCs (Fig. 2*G*, *H*, *I*). The iPSCs are free from mycoplasma contamination. Short tandem repeat (STR) analysis results showed that CS-iPSCs were identical to CS patient PBMC (Supplementary data 2*A*). Karyotype analysis revealed that CS-iPSCs were 46, XX (Supplementary data 2*B*).

### **PTEN expression decreased and AKT signaling–related gene expression increased**

**Fig. 3:**
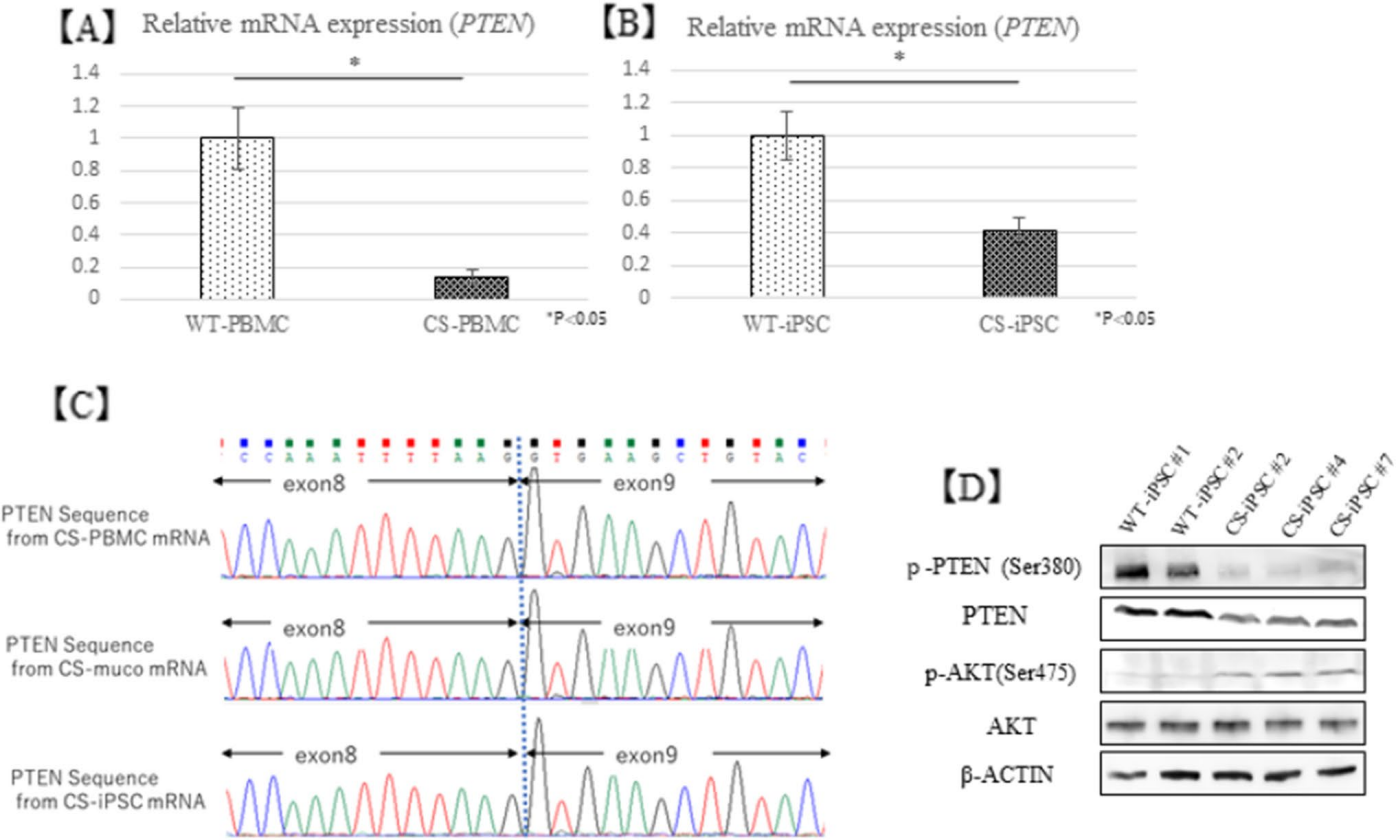

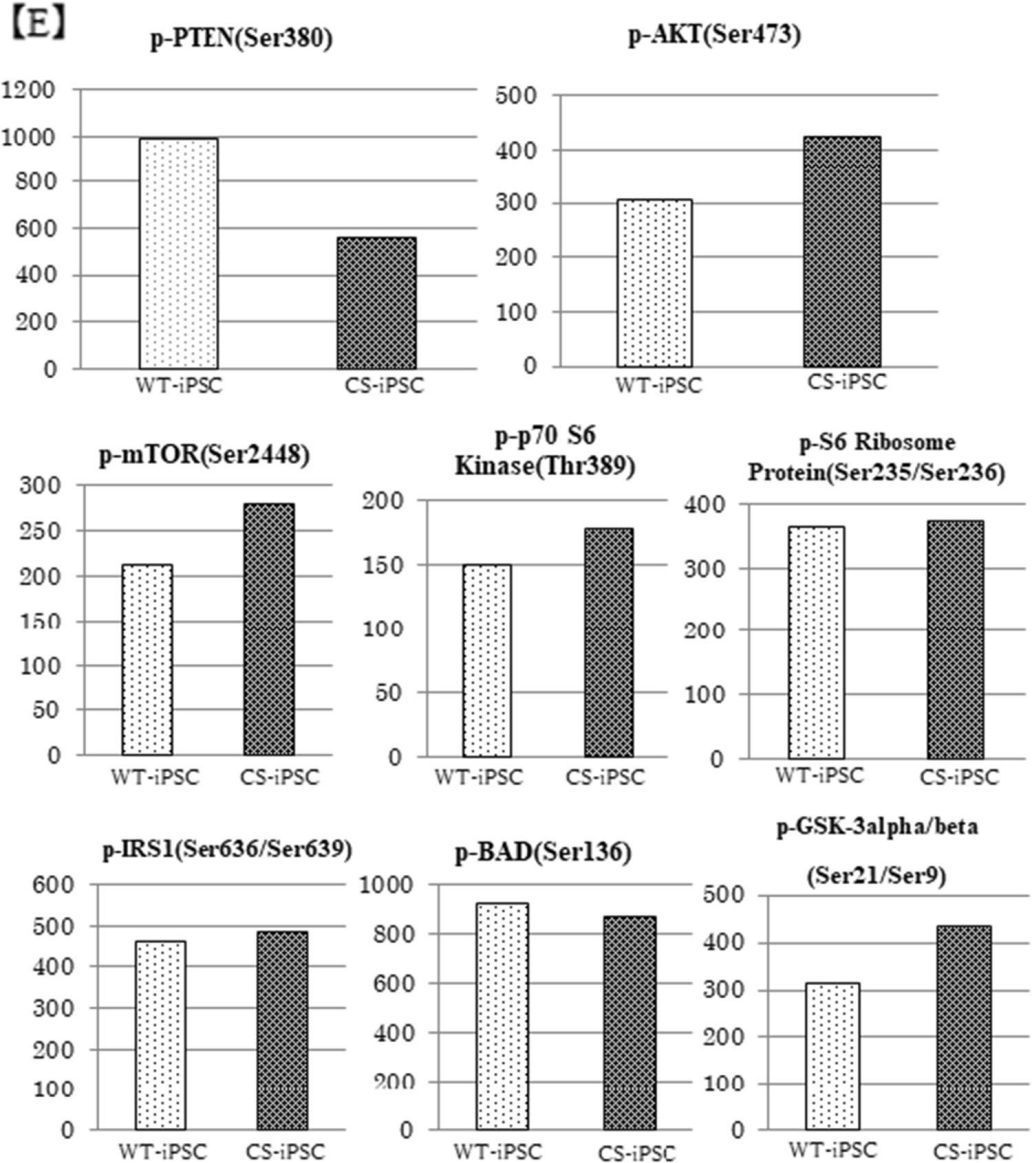
PTEN gene expression and PTEN/AKT pathway activity. (*A*, *B*) PTEN mRNA expression in CS-PBMC and -iPSC. (*C*) PTEN mRNA sequencing by the Sanger method. (*D*) PTEN and AKT protein expression in CS-iPSC. (*E*) Analysis of AKT signaling activity by the Bio-Plex assay.

Gene expression of *PTEN* was significantly decreased in CS-iPSCs, compared with WT-iPSCs (Fig. 3*A*). We also observed a reduction in *PTEN* gene expression in CS-PBMCs (Fig. 3*B*). The mRNA extracted from CS-PBMCs and CS-iPSCs was sequenced by the Sanger method using primers set from exon 7 to exon 9 of *PTEN* (Table 1), but the transcript from the mutant allele was not observed (Fig. 3*C*). Analysis of the phosphorylation levels of AKT signaling–related proteins by western blotting and Bio-Plex systems showed that phosphorylated PTEN was decreased, while the phosphorylation levels of AKT, mTOR, and GSK3α/β were increased (Fig. 3*D*, *E*).

### **Expression of PTENδ derived from the mutant allele increased in CS-iPSC**

**Fig. 4:**
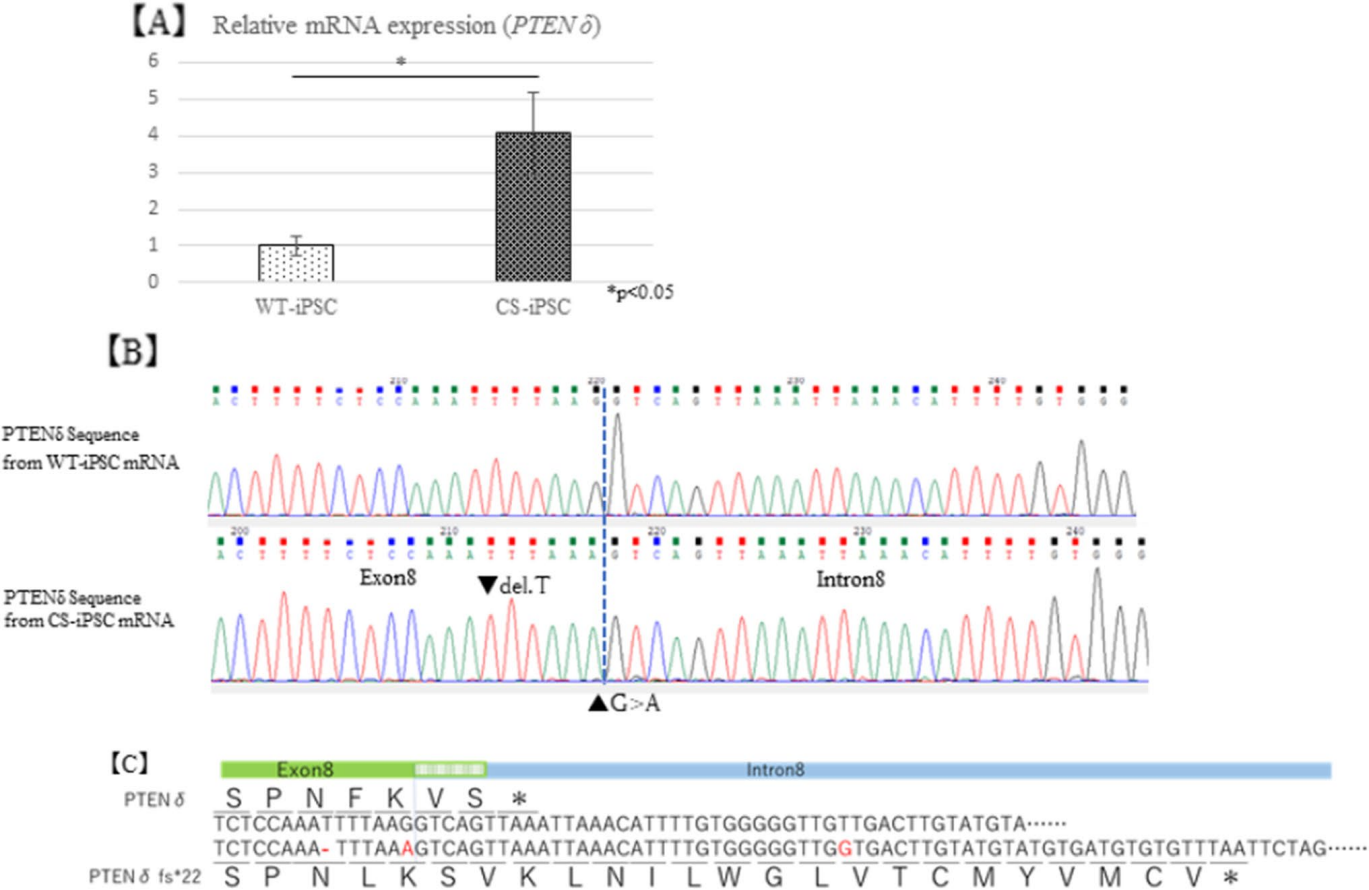
PTEN δ expression in iPSCs. (*A*) PTEN δ mRNA expression in CS-iPSC was higher than that in WT-iPSCs. (*B*) PTEN δ sequencing from iPSCs by the Sanger method. (*C*) Predicted protein sequence variant of these mutations.

To analyze the expression of *PTEN δ*, the *PTEN* splicing variant, we designed primers for *PTEN* exon 7 and *PTEN* intron 8, sequenced them by the Sanger method, and analyzed their expression levels using RT-qPCR. Interestingly, the expression of *PTEN δ* was significantly upregulated in CS-iPSCs (Fig. 4*A*), and there was no expression from the wild-type allele (Fig. 4*B*).

## Discussion

In this case study, the patient had numerous polyps in the oral mucosa, trichilemmomas on the face, and macrocephaly. She also had a medical history of breast cancer and thyroid cancer. Although acral keratosis, autism spectrum disorder, and developmental delay were not found, her symptoms met the typical clinical criteria of CS (Milella *et al.*, 2015). These symptoms led us to search for mutations of *PTEN*.

First, we examined the whole exon region of *PTEN* by the single-strand conformation polymorphism method. However, we found no results suggesting mutation. NGS analysis revealed two germline mutations (c.1020delT and c.1026G > A) at the end of *PTEN* exon 8. Since these mutations were located at the end of exon 8 and did not cause remarkable changes in the higher-order structure of the DNA, they could not be detected by the Polymerase Chain Reaction-Single-Strand Conformation Polymorphism method. Evaluation of the splicing sites showed that splicing was likely affected by these mutations (Table 4). Recently, Chen *et al.*. reported that some variants of *PTEN* result in pathogenic exon skipping, alternative splicing, or use of cryptic splice sites in CS (Chen *et al.*, 2017). It is known that mRNAs containing incorrect translation termination codons, caused by errors during transcription and replication or splicing, are selectively degraded by the nonsense-mediated mRNA decay pathway (Behm-Ansmant *et al.*, 2007). Reduction in *PTEN* gene expression and loss of the transcript from the mutant allele may indicate that this mutation causes intron retention and aberrant mRNA degradation by the NMD pathway. Moreover, analysis of *PTEN* gene expression in buccal mucosal tumors of patients showed that it did not cause obvious homozygous loss. These findings support that the pathogenesis was caused by haploinsufficiency as has been reported (Di Cristofano *et al.*, 1999, 2001; Salmena *et al.*, 2008). In addition, an intron mutation (c.1026 + 32 T > G) has been suggested to affect the splicing silencer site in the exon, but this mutation has a high allele frequency, and according to ClinGen PTEN Expert Panel Specifications, it has a benign stand-alone effect (Mester *et al.*, 2018).

**Table 4:** Analysis result of spicing site variant using human splicing finder.

PTEN mutation	Predicted signal	Predicted algorithm	Interpretation
c.1020delT			Probably no impact on splicing
c.1026G > A	Broken splicing donor site	HSF Matrices MaxEnt	Alteration of splicing donor site most probably affecting splicing
c.1026 + 321 > G	ESS site broken	Fas-ESS hexamers	Alteration of an intronic splicing silencer site

The advantages of using iPSCs to study diseases of hereditary tumor syndromes include solving the problem of an insufficient supply of samples from specimens, as well as producing cells that cannot be established as cell lines by differentiating into specific cells. Furthermore, it may be useful for tracing the mechanism of tumorigenesis. The CS-iPSCs established in this study showed reduced *PTEN* gene expression and protein expression, compared to WT-iPSCs. The CS-iPSCs also showed high induction efficiency. However, the effects on the AKT pathway due to *PTEN* reduction seemed to be corrected downstream, and we did not observe the phenotype such as hamartoma or neoplastic growth that this patient developed in clinical practice. It may be that haploinsufficiency of *PTEN* alone is not sufficient for the development of CS, and additional factors are required.

There are several variants of *PTEN*, including a variant containing intron 8 (*PTEN δ*) (Sharrard and Maitland, 2000). The function of PTEN δ is reported to be similar to that of PTEN (Breuksch *et al.*, 2018), but the details are unknown. We analyzed the expression level and nucleotide sequence of *PTEN δ* by RT-qPCR and Sanger sequencing using the primers designed for exon 7 and intron 8. Gene expression of *PTEN δ* was low in WT-PBMCs and CS-PBMCs, making it difficult to compare the two. However, compared to WT-iPSCs, the expression of *PTEN δ* in CS-iPSCs was predominantly high, and the expression from the wild-type allele was suppressed. This suggests that abnormal translation from intron 8 causes structural changes in the C-terminus of *PTEN δ* (Fig. 4*C*) and aberrant *PTEN δ* may be involved in the pathogenesis of this disease.

Using NGS, we also discovered other germline genetic variants associated with tumor development. *MLH1* is a component of intracellular DNA mismatch repair (MMR) and is also known to be involved in genomic instability and Lynch syndrome. V384D of *MLH1* is a polymorphism found in approximately 2.5% of East Asians, and this variant is known to have reduced MMR activity. The V384D variant of *MLH1* is frequently found in breast cancer tissues, suggesting that haploinsufficiency of *MLH1* may modulate malignant tumor progression (Lee *et al.*, 2019). The *MSH6* gene is also involved in genomic stability, and it has been suggested that G39E may be associated with breast cancer (Lee *et al.*, 2014), but no conclusion has been reached yet. Furthermore, *BRCA2* is known as a causative gene of hereditary breast and ovarian cancers, but mutant M784V has been reported to be neutral (Spearman *et al.*, 2008). The relationship between these mutations and *PTEN* has not been fully elucidated, but mutations in these genes may support the patient’s medical history.

## Conclusion

Although there are many reported cases of Cowden syndrome, a few studies have focused on its pathogenesis. In this study, we report a novel mutation of *PTEN* and the establishment of the first CS-iPSCs, and analyzed the expression of the *PTEN* gene. In recent years, with the progress of cancer genome medicine, the identification of hereditary tumor-associated genes such as *PTEN* has been progressing. However, further studies on pathogenic mutations and their pathogenesis are required to establish a comprehensive therapeutic system for hereditary tumors. Using CS disease-specific iPSCs, as shown in this study, will provide further new insights for disease research and therapeutic drug discovery.

## Supplementary Information

Below is the link to the electronic supplementary material.

Supplementary file1 (DOCX 1193 KB)

## References

[CR1] Behm-Ansmant I, Kashima I, Rehwinkel J, Saulière J, Wittkopp N, Izaurralde E (2007). mRNA quality control: an ancient machinery recognizes and degrades mRNAs with nonsense codons. FEBS Lett.

[CR2] Breuksch I, Welter J, Bauer HK, Enklaar T, Frees S, Thüroff JW, Hasenburg A, Prawitt D, Brenner W (2018). In renal cell carcinoma the PTEN splice variant PTEN-Δ shows similar function as the tumor suppressor PTEN itself. Cell Commun Signal.

[CR3] Chen HJ, Romig T, Sesoc K, Eng C (2017). Characterization of cryptic splicing in germline PTEN intronic variants in Cowden syndrome. Hum Mutat.

[CR4] Di Cristofano A, De Acetis M, Koff A, Cordon-Cardo C, Pandolfi P (2001). Pten and p27KIP1 cooperate in prostate cancer tumor suppression in the mouse. Nat Genet.

[CR5] Di Cristofano A, Kotsi P, Peng YF, Cordon-Cardo C, Elkon KB, Pandolfi PP (1999). Impaired Fas response and autoimmunity in Pten+/- mice. Science.

[CR6] Eng C (1997). Cowden Syndrome. J Genet Couns.

[CR7] Eng C (2000). Will the real Cowden syndrome please stand up: revised diagnostic criteria. J Med Genet.

[CR8] Hamada A, Akagi E, Obayashi F, Yamasaki S, Koizumi K, Ohtaka M, Nishimura K, Nakanishi M, Toratani S, Okamoto T (2020). Induction of Noonan syndrome-specific human-induced pluripotent stem cells under serum-, feeder-, and integration-free conditions. In Vitro Cell Dev Biol Anim.

[CR9] Hamada A, Akagi E, Yamasaki S, Nakatao H, Obayashi F, Ohtaka M, Nishimura K, Nakanishi M, Toratani S, Okamoto T (2020). Induction of integration-free human-induced pluripotent stem cells under serum- and feeder-free conditions. In Vitro Cell Dev Biol Anim.

[CR10] Lee E, Levine EA, Franco VI, Allen GO, Gong F, Zhang Y, Hu JJ (2014). Combined genetic and nutritional risk models of triple negative breast cancer. Nutr Cancer.

[CR11] Lee SE, Lee HS, Kim KY, Park JH, Roh H, Park HY, Kim WS (2019). High prevalence of the MLH1 V384D germline mutation in patients with HER2-positive luminal B breast cancer. Sci Rep.

[CR12] Mester JL, Ghosh R, Pesaran T, Huether R, Karam R, Hruska KS, Costa HA, Lachlan K, Ngeow J, Barnholtz-Sloan J (2018). Gene-specific criteria for PTEN variant curation: recommendations from the ClinGen PTEN Expert Panel. Hum Mutat.

[CR13] Milella M, Falcone I, Conciatori F, Cesta Incani U, Del Curatolo A, Inzerilli N, Nuzzo CM, Vaccaro V, Vari S, Cognetti F (2015). PTEN: Multiple functions in human malignant tumors. Front Oncol.

[CR14] NCCN.org (2019) NCCN Clinical practice guilines in oncology genetic/familial high-risk assessment: breast and ovarian,and pancreatic. Version,2021. [(accessed on 19 November 2020)] https://www.nccn.org/professionals/physician_gls/pdf/genetics_bop.pdf

[CR15] Salmena L, Carracedo A, Pandolfi PP (2008). Tenets of PTEN tumor suppression. Cell.

[CR16] Sato JD, Kawamoto T, Okamoto T (1987). Cholesterol requirement of P3–X63-Ag8 and X63-Ag8.653 mouse myeloma cells for growth in vitro. J Exp Med.

[CR17] Sharrard RM, Maitland NJ (2000). Alternative splicing of the human PTEN/MMAC1/TEP1 gene. Biochim Biophys Acta.

[CR18] Spearman AD, Sweet K, Zhou XP, McLennan J, Couch FJ, Toland AE (2008). Clinically applicable models to characterize BRCA1 and BRCA2 variants of uncertain significance. J Clin Oncol.

[CR19] Takahashi K, Tanabe K, Ohnuki M, Narita M, Ichisaka T, Tomoda K, Yamanaka S (2007). Induction of pluripotent stem cells from adult human fibroblasts by defined factors. Cell.

[CR20] Takahashi K, Yamanaka S (2006). Induction of pluripotent stem cells from mouse embryonic and adult fibroblast cultures by defined factors. Cell.

[CR21] Tan MH, Mester JL, Ngeow J, Rybicki LA, Orloff MS, Eng C (2012). Lifetime cancer risks in individuals with germline PTEN mutations. Clin Cancer Res.

[CR22] Yamasaki S, Taguchi Y, Shimamoto A, Mukasa H, Tahara H, Okamoto T (2014). Generation of human induced pluripotent stem (Ips) cells in serum- and feeder-free defined culture and TGF-Β1 regulation of pluripotency. PLoS ONE.

