# Supplementary material for: Identification of a Cowden syndrome patient with a novel PTEN mutation and establishment of patient-derived induced pluripotent stem cells
Source: In Vitro Cell Dev Biol Anim. 2022 Jan 3;58(1):69–78. doi: 10.1007/s11626-021-00637-8 (PMC8803725; doi:10.1007/s11626-021-00637-8)
Supplement: Supplementary file 1 — Supplementary file1 (DOCX 1193 KB) [file 11626_2021_637_MOESM1_ESM.docx]

**Supplementary Information**

**Identification of a Cowden syndrome patient with a novel *PTEN* mutation and establishment of patient-derived induced pluripotent stem cells**

Fumitaka Obayashi^1^, Atsuko Hamada^1^, Sachiko Yamasaki^1^, Taku Kanda^3^, Shigeaki Toratani^2^, Tetsuji Okamoto^2,4^

^1^ Oral and Maxillofacial Surgery, Hiroshima University Hospital, Hiroshima, Japan

^2^ Department of Molecular Oral Medicine and Maxillofacial Surgery, Graduate School of Biomedical and Health Science, Hiroshima University, Hiroshima, Japan

^3^Oral and maxillofacial Surgery, Hiroshima Prefectural Hospital, Hiroshima, Japan

^4^School of Medical Sciences, The University of East Asia, Shimonoseki 751-8503, Yamaguchi, Japan

Journal name: In Vitro Cellular & Developmental Biology - Animal

**Correspondence**: Tetsuji Okamoto　 Email address: tetsuok@hiroshima-u.ac.jp

**Correspondence**: Atsuko Hamada　 E-mail: hamaco@hiroshima-u.ac.jp

Department of Molecular Oral Medicine and Maxillofacial Surgery, Division of Applied Life Science, Graduate Institute of Biomedical and Health Science, Hiroshima University,

1-2-3, Kasumi, Minami-ku, Hiroshima-city, Hiroshima 734-8553, Japan


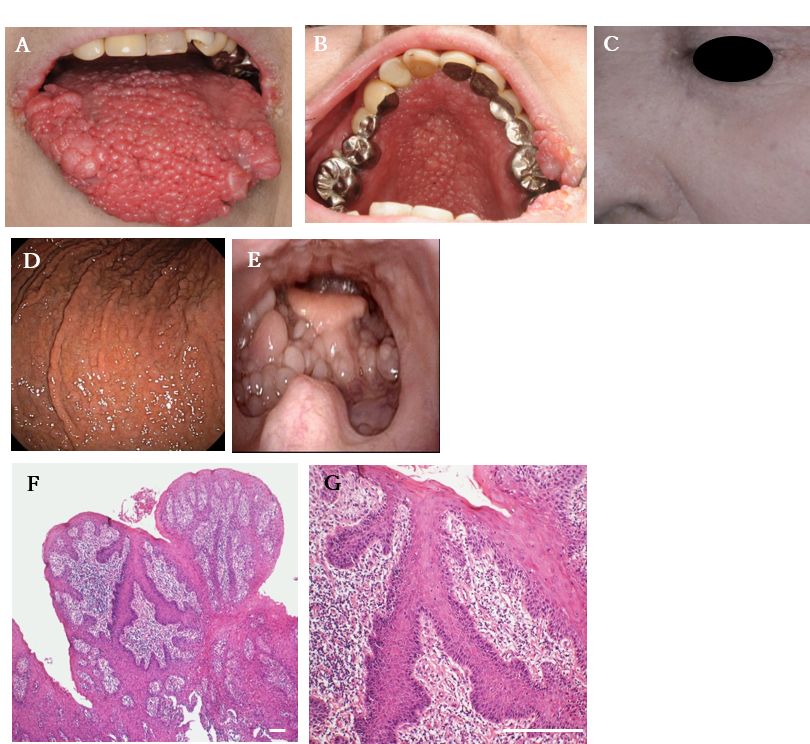


Supplementary data 1. (A, B): Cobblestone-like lesions over the tongue (A) and palate (B). (C) Keratotic papules on facial skin. (D, E): Fiber scope showing multiple polyps in the pharynx (D) and gastrointestinal tract (E). (F, G): Histological findings reveal verrucous mucosal hyperplasia in the buccal mucosa. (Scale bar,100µm)


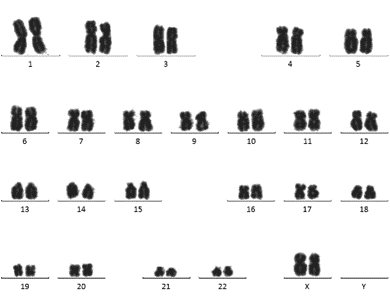


【A】　CS-iPSC passage 48


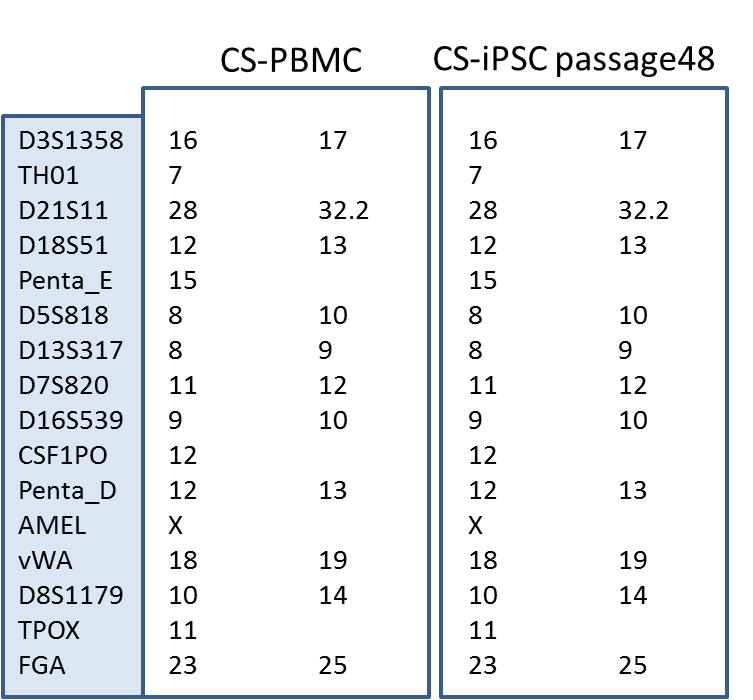


【B】

CS-iPSC passage48

CS-PBMC

Supplementary data 2. Karyotype analysis of CS-iPSCs passage 48 (A). STR analysis of CS-iPSCs (B).
